# AFNet Algorithm for Automatic Amniotic Fluid Segmentation from Fetal MRI

**DOI:** 10.3390/bioengineering10070783

**Published:** 2023-06-30

**Authors:** Alejo Costanzo, Birgit Ertl-Wagner, Dafna Sussman

**Affiliations:** 1 Department of Electrical, Computer and Biomedical Engineering, Faculty of Engineering and Architectural Sciences, Toronto Metropolitan University, Toronto, ON M5B 2K3, Canada; 2 Institute for Biomedical Engineering, Science and Technology (iBEST), Toronto Metropolitan University and St. Michael’s Hospital, Toronto, ON M5B 1T8, Canada; 3 Department of Diagnostic Imaging, The Hospital for Sick Children, Toronto, ON M5G 1X8, Canada; 4 Department of Medical Imaging, Faculty of Medicine, University of Toronto, Toronto, ON M5T 1W7, Canada; 5 Department of Obstetrics and Gynecology, Faculty of Medicine, University of Toronto, Toronto, ON M5G 1E2, Canada

**Keywords:** medical image segmentation, amniotic fluid, AFNet, fetal MRI, Magnetic Resonance Imaging, CNN, deep learning

## Abstract

Amniotic Fluid Volume (AFV) is a crucial fetal biomarker when diagnosing specific fetal abnormalities. This study proposes a novel Convolutional Neural Network (CNN) model, AFNet, for segmenting amniotic fluid (AF) to facilitate clinical AFV evaluation. AFNet was trained and tested on a manually segmented and radiologist-validated AF dataset. AFNet outperforms ResUNet++ by using efficient feature mapping in the attention block and transposing convolutions in the decoder. Our experimental results show that AFNet achieved a mean Intersection over Union (mIoU) of 93.38% on our dataset, thereby outperforming other state-of-the-art models. While AFNet achieves performance scores similar to those of the UNet++ model, it does so while utilizing merely less than half the number of parameters. By creating a detailed AF dataset with an improved CNN architecture, we enable the quantification of AFV in clinical practice, which can aid in diagnosing AF disorders during gestation.

## 1. Introduction

Amniotic fluid is a vital biological fluid necessary for the development of the fetus. It is an extracellular fluid in the amniotic sac surrounding the fetus [1,2,3]. The fluid is crucial in facilitating fetal lung development, swallowing, skeletal movement, and regulating temperature and anti-inflammatory functions [3]. Throughout gestation, various dynamic processes, such as fetal breathing and swallowing, regulate amniotic fluid [2]. Disruptions in these dynamic processes can result in low amniotic fluid volume (oligohydramnios) or high amniotic fluid volume (polyhydramnios), which occur in approximately 1–2% of pregnancies due to underlying causes and are associated with poor pregnancy outcomes [1,3,4,5,6,7,8].

Quantifying amniotic fluid volume is often challenging using non-invasive fetal imaging techniques, primarily ultrasound (US). The Single Deepest Pocket (SDP) and Amniotic Fluid Index (AFI) are the most commonly employed ultrasound-based techniques [2,3,4,5,6,7]. While SDP and AFI can reasonably estimate amniotic fluid volume (AFV) disorders [8], they lack precision in volumetric measurements. AFV is estimated by SDP. Consequently, their specificity is low (<32%) in cases of high or low AFV [8,9]. Dye-dilution methods and direct measurement during a Cesarean delivery are currently the most accurate techniques for estimating AFV [10]. However, these invasive methods can only establish statistical correlations between AFV and estimation techniques without directly relating them to clinical outcomes [10].

Fetal Magnetic Resonance Imaging (MRI) offers high soft tissue contrast compared to ultrasound [5,11,12,13] and provides more comprehensive information [14] for diagnosing specific fetal or maternal abnormalities, such as oligohydramnios or polyhydramnios. The use of MRI in pregnancies is considered safe by the Canadian Association of Radiologists, particularly after the first trimester and without gadolinium-based contrast agents [15]. With its superior contrast, spatial resolution, and ability to cover the uterus, fetal MRI enables quantitative assessment of the entire amniotic fluid volume. This assessment aids in diagnostic and therapeutic decision-making as well as clinical research. However, the manual segmentation of amniotic fluid volume on MRI sequences is highly burdensome, time-consuming, and impractical for routine clinical assessments.

The accurate and complete segmentation of amniotic fluid (AF) is essential for obtaining the total amniotic fluid volume (AFV) from MRI. Segmentation involves labelling each pixel in an image, typically done manually by expert radiologists trained to differentiate between the target label and the image background. This process is time-consuming and costly, requiring the segmentation of hundreds of images for each patient volume [16,17]. To address these challenges and improve segmentation’s speed, accuracy, and cost-effectiveness, machine learning techniques that can perform comparably to experts have been implemented [16,17,18]. Previous machine learning tools have only focused on automatic segmentation of ultrasound (US) images [19,20,21]; yet, the increased reliance on fetal MRI emphasizes the need for such tool development for MRI applications too.

Machine learning, specifically deep learning segmentation models, has revolutionized medical image analysis, offering accurate and efficient automated segmentation of anatomical structures in MRI scans [22,23,24,25,26,27,28,29,30]. Convolutional neural networks (CNNs) are the foundation for these models, as they can learn complex features from images. The network is trained by optimizing parameters across multiple layers of convolutional filters, normalization layers, and dense layers to minimize the error (loss) on the training dataset. Various techniques, including skip connections [31], attention mechanisms [32,33], transpose convolutions [34], and atrous convolutions [29,35] are incorporated into many architectures to improve network performance. Due to the variability in CNN models, refining existing architectures through small changes in hyperparameters, model size, and model layers is crucial for achieving optimal performance. Previous studies have demonstrated strong model performance on the fetal brain [36], placental [37], and body [38] segmentations using MRI datasets.

There is a lack of literature on applying deep learning models to segment amniotic fluid from MRI. Existing US-based models are not applicable due to significant differences in image domains. The segmentation of AF is challenging due to the presence of structures that may have similar pixel intensities and can be mistaken as amniotic fluid. This study aims to introduce an expert-validated MRI dataset with segmented amniotic fluid. It proposes a novel architecture called AFNet, which outperforms state-of-the-art medical segmentation networks on our dataset. Our model enables automated quantification of amniotic fluid volume using fetal MRI datasets, opening avenues for further research to enhance clinical outcomes related to amniotic fluid-related disorders.

## 2. Methods

### 2.1. Dataset

For this study, the dataset used consisted of 45 T2-weighted 3D fetal MRI sequences obtained using an SSFP sequence on a 1.5 T or a 3.0 T MR scanner. In this dataset, we obtained 2D coronal reformatted images, with each patient having between 50–120 slices. We resized varying 2D image slices along the frontal to 512 × 512, and each slice was individually intensity normalized. The prediction of AF on each 2D T2-weighted MRI slice can then be used to obtain the full 3D rendering of AF. The local research ethics board approved the use of patient data for this study, considering that all data be de-identified and not contain any rare (1:10,000) pathological features. Manual segmentation of the amniotic fluid was performed in-house with the aid of the segmentation software Amira-Avizo (Berlin, Germany), and then verified by an expert radiologist. The amniotic fluid was segmented on each slice in the frontal plane, using contrast thresholding on the magic wand, lasso and brush tools for clear segmentation boundaries. Due to legal restrictions on our medical data, this dataset can not be made publicly available.

### 2.2. Model Architecture

**Figure 1:**
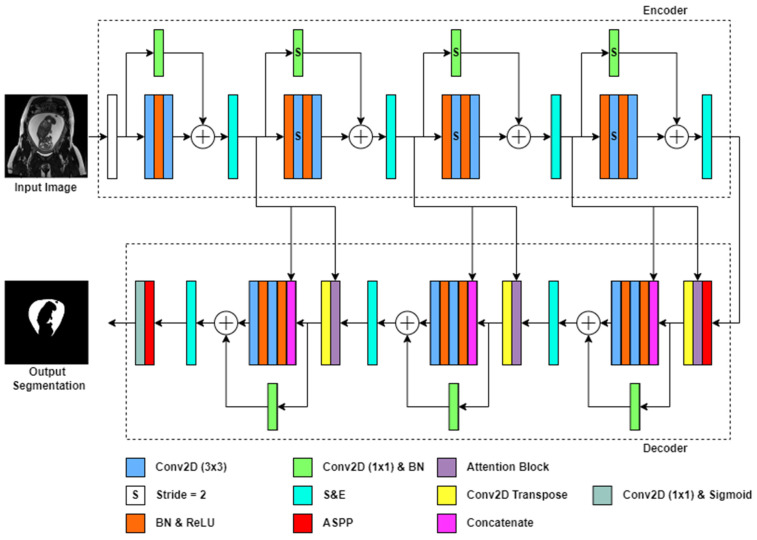
AFNet architecture. Rectangles represent different blocks or functions in the network, while arrows represent the data flow. A legend is shown below the network to colour–code the differing model blocks. Unless otherwise indicated, the stride is 1 for convolutional layers. A fetal MRI input image of 512 × 512 feeds into the network and outputs a segmentation mask for amniotic fluid.

We present a novel architecture called AFNet, inspired by the original ResUNet++ [24], as shown in Figure 1. Our modifications to ResUNet++ enhance the network’s performance by refining and improving its existing layers, specifically tailored to the challenges of medical segmentation. We chose this architecture due to its competitive results on the complex medical segmentation datasets of polyp segmentation [39,40]. Therefore, we chose it as our foundation due to its proven performance and ease of implementation. However, we recognized the need for further advancements to address the specific requirements of our task.

The original architecture follows an encoder-decoder structure. We devised the ResUNet++ encoder to comprise four residual blocks in series. The first residual block, the stem, has two 3 × 3 convolutional layers with a stride of 1, a batch normalization (BN) layer, a ReLU activation layer, a residual connection, and a squeeze & excitation layer. The subsequent three residual blocks are similar to the stem, except the first convolutional layer has a stride of 2, the skip connection convolutional layer has a stride of 2, and an additional BN and ReLU layer. These modifications enable an effective latent space representation of the image through atrous convolutions.

At the decoder stage, the image dimensionality is reduced by a factor of 8. This stage reconstructs the image from high-level features in the latent space representation of the image. A decoder block consists of three residual blocks, an attention block, and a transpose convolutional layer to double the image dimensionality. Furthermore, we incorporated an atrous spatial pyramid pooling (ASPP) block at the beginning and end of the decoder stage, along with a 1 × 1 convolutional layer and a sigmoid activation layer. Skip connections are present after each residual block in the encoder stage, allowing the network to retain important feature mappings in the residual connection. Notably, we replaced the traditional upsampling layers with transposed convolutional layers [41], contributing to the uniqueness of AFNet and facilitating robust feature extraction during upsampling. In addition to architectural changes, we fine-tuned and refined the attention block to better suit the requirements of our modified network. These refinements significantly enhance the overall performance and capabilities of AFNet.

The Atrous Spatial Pyramid Pooling (ASPP) module, as discussed in [29,35], has proven to be effective in capturing multi-scale information for segmentation tasks. It consists of multiple parallel atrous convolutional layers at different rates, allowing for capturing both local and global information. The ASPP module bridges the encoder and decoder stages, facilitating the extraction, creation, and enhancement of deep feature maps within the encoder at various receptive layers.

The ASPP module comprises five parallel paths. Each path includes a convolutional layer, followed by batch normalization and a ReLU activation layer. The first path employs a 1 × 1 convolutional layer, while the remaining three paths utilize 3 × 3 convolutional layers with atrous rates of 6, 12, and 18, respectively. Additionally, there is a path dedicated to capturing global features. It involves an average pooling layer, followed by a 1 × 1 convolutional layer, batch normalization, ReLU activation, and resizing the image back to its original dimension. Finally, the outputs from all paths are concatenated and passed through another set of convolutional layers, batch normalization, and ReLU activation.

The ASPP module effectively integrates multi-scale information into the segmentation process. Incorporating parallel convolutional layers with different atrous rates and a path for capturing global features enhances the network’s performance in fully connected segmentation tasks. The module is crucial in extracting and highlighting deep feature maps within the encoder, contributing to improved segmentation accuracy.

#### Attention Block

Our study introduces a novel attention block that modifies the existing attention block used in the ResUNet++ model [24]. This modification aims to enhance the interaction between the encoder and decoder layers in our architecture. In our framework, we consider the output encoder feature map $E^{\left[e\right]}$, where e represents the number of encoder blocks the feature map has passed. Similarly, the decoder feature map $D^{\left[d\right]}$, where d corresponds to the number of decoder blocks the feature map has traversed, initially starting at zero. The relationship between the encoder and decoder layers can be described using Equation (1), where B denotes the total number of encoder blocks excluding the stem block.

Unlike the original ResUNet++ architecture, the input encoder feature map in our attention block has twice the spatial dimensions (H×W) compared to the decoder feature map $D^{\left[d\right]}\in R^{H\times W\times C}$ with C representing the number of channels. By concatenating the encoder feature maps onto the decoder, our network can effectively extract low-level spatial features and integrate them with abstract high-level decoder feature maps, facilitating fine-grain segmentation.

To process the input feature map $X^{\left[l\right]}$ at layer l, which includes a weight matrix $W^{\left[l\right]}$, bias matrix $b^{\left[l\right]}$, and activation function g, we employ a convolutional layer, as depicted in Equation (2). The function $f_{s}$ is introduced to incorporate an atrous convolution of stride 2, while batch normalization is omitted for clarity. As a result, the output decoder matrix $D_{o}^{\left[d\right]}$ generated by the attention block, as described in Equation (3), contains a finely calibrated feature map that integrates the encoder and decoder information.

(1) d=−e+B

(2) $$f(X^{\left[l-1\right]})=W^{\left[l\right]}*g(X^{\left[l-1\right]})+b^{\left[l\right]}=X^{\left[l\right]}$$

(3) $$D_{o}^{\left[d\right]}=f(f_{s}(f(E^{\left[e\right]}))+f(D^{\left[d\right]}))\cdot D^{\left[d\right]}$$

**Figure 2:**
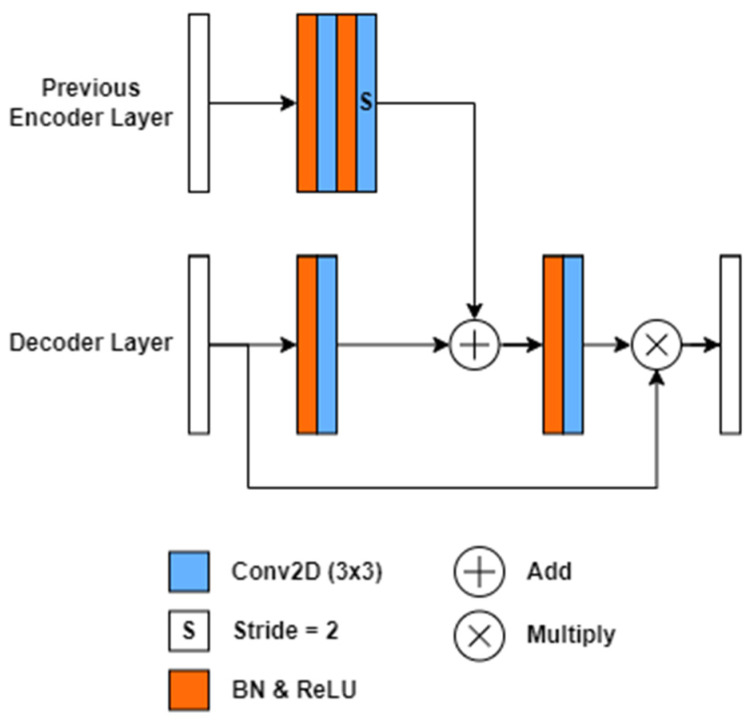
Proposed attention block architecture used in AFNet. A legend is shown below the network to colour–code the differing model blocks. Unless otherwise indicated, the stride is 1 for convolutional layers.

Our attention mechanisms play a crucial role in improving the feature maps of specific areas within the network by establishing modified connections with previous layers. Figure 2 showcases the attention block incorporated into our modified ResUNet++ architecture. Notably, a max pooling layer was employed in the original encoder attention path, while our proposed attention mechanism replaces it with an atrous convolution block of stride two. This modification enables more efficient and representative encoding within the decoder layer, leveraging the valuable information from previous encoder feature maps.

Introducing these novel attention mechanisms and adapting the ResUNet++ architecture enhances the interaction between encoder and decoder layers, leading to improved performance and more efficient encoding in our modified framework.

### 2.3. Evaluation Metrics

To evaluate the performance of our deep learning model, we employ essential evaluation metrics that provide insights into the accuracy of the network when presented with unseen data. In semantic segmentation tasks, the F1 score (Dice Coefficient) and the *Jaccard* index (intersection over union) are widely used metrics to quantify the agreement between the predicted segmentation maps and the ground truth [42].

Let us denote the ground truth segmentation dataset as *S_g_* and the network’s output segmentation as *S_o_*. The F1 score and *Jaccard* index are defined by Equations (4) and (5), respectively. These metrics enable us to measure the degree of overlap between the predicted and ground truth segmentation maps.

In addition to the F1 score and *Jaccard* index, we consider recall and precision metrics in our model comparison. These metrics provide further insights into the model’s performance. However, they become more meaningful when the mean intersection over union (mIoU) scores are statistically insignificant among the compared models. The mIoU represents the average intersection over union across the entire dataset, providing a comprehensive measure of segmentation accuracy.

(4) $$Jaccard=IoU=\frac{\left|S_{g}\cap S_{o}\right|}{\left|S_{g}{\cup S}_{o}\right|}=\frac{TP}{TP+FP+FN}$$

(5) $$Dice=\frac{2\left|S_{g}\cap S_{o}\right|}{\left|S_{g}\right|+\left|S_{o}\right|}=\frac{2TP}{2TP+FP+FN}=\frac{2Jaccard}{Jaccard+1}$$

In our analysis, we incorporate the recall metric, also known as the True Positive Rate (6), to assess the model’s performance in amniotic fluid segmentation, with a focus on capturing all instances of the target class. This metric helps us evaluate how well the model identifies and includes relevant regions of the amniotic fluid in the segmentation output, considering the potential for over-segmentation.

Additionally, we utilize the precision metric (7) to evaluate the model’s performance, specifically in under-segmenting the amniotic fluid, aiming to minimize false positives. Precision measures the proportion of correctly identified amniotic fluid regions out of all the predicted positive regions. By considering both recall and precision, we comprehensively understand the model’s ability to balance between over-segmentation and under-segmentation in amniotic fluid segmentation [43].

Medical segmentation tasks often require striking a balance between capturing all relevant regions (avoiding false negatives) and minimizing false positives, as defined in Table 1. The combination of recall and precision metrics provides a robust evaluation framework less sensitive to predictions exhibiting over-segmentation and under-segmentation tendencies. By utilizing these metrics, we can effectively assess the model’s performance and ability to achieve accurate and balanced amniotic fluid segmentation results.

(6) $$Precision=\frac{TP}{TP+FP}$$

(7) $$Recall=\frac{TP}{TP+FN}$$

### 2.4. Training Implementation

**Figure 3:**
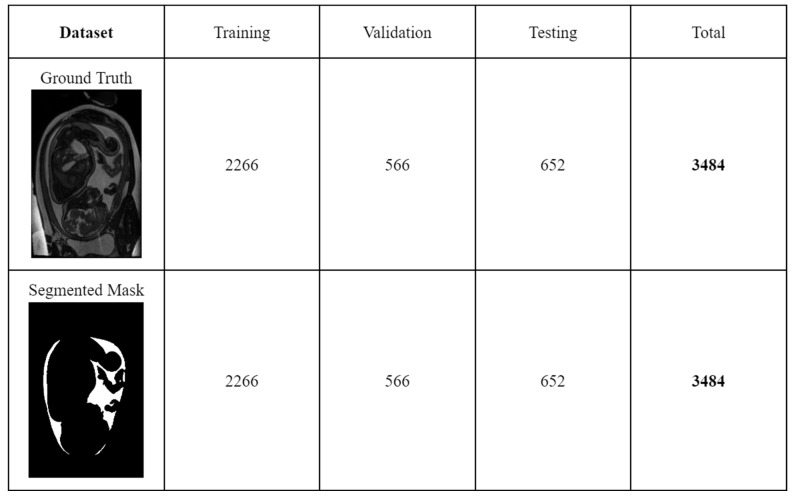
Dataset division for training, validation, and testing.

The dataset was split into the train, validation, and test sets using a ratio of 65/15/20 per cent, respectively, after randomly shuffling the entire dataset giving Figure 3. The holdout test set was exclusively used once for evaluating the models after fine-tuning them on the validation set. This approach ensures unbiased performance metrics [44].

Normalization was applied to the training set, scaling the pixel values to the range of [0, 1]. Data augmentation techniques were employed in the training set to enhance image diversity and promote model generalizability. These techniques included random image contrast adjustments within the range of [0.4, 0.6) and random flips.

To accommodate the entire image within the model while staying within memory limitations, the image slices were resized to 512 × 512 dimensions. Hyperparameter tuning was performed empirically, and it was found that the same set of hyperparameters could be used for most networks. However, for AFNet and ResUNet++, better performance was achieved using the Adagrad optimizer with a higher learning rate of 0.1 and gradient clipping. On the other hand, the Adam optimizer demonstrated superior performance with other state-of-the-art networks, using a learning rate of 0.0001. Attempts to use higher learning rates with Adam did not yield optimal results for the other networks. The batch size hyperparameter was determined to be equally optimal for all networks.

An exponential decay factor with a decay rate of 0.96 and decay steps of 10,000 was applied to control the learning rate. The network was trained for a maximum of 200 epochs with a batch size of 8. Early stopping was implemented using a callback mechanism, monitoring the mean Intersection over Union (*IoU*) metric with a patience of 10 epochs and a baseline of 0.75. As a result, most networks converged within 50 to 150 epochs. The dice loss function was chosen due to its robustness in training networks to recognize similarities in the ground truth.

The implementation of this model utilized TensorFlow, and training was performed on Compute Canada’s Cedar cluster network. A single node comprising two Intel Silver 4216 Cascade Lake processors running at 2.1 GHz and four NVIDIA V100 Volta GPUs (each with 32 GB of HBM2 video memory) was utilized for training.

## 3. Results & Discussion

We conducted a series of experiments to evaluate the performance of our AFNet model and determine any statistically significant improvements compared to other models. The models included the original ResUNet++ model proposed in [24], ResUNet++ with only the modified attention and no transposed convolution (AFNet noT), and ResUNet++ with both the modified attention and transpose convolutions (AFNet). These models were trained using the same optimized hyperparameters and training scheme. Each model underwent approximately 30 training runs with random weight initialization on the same dataset split. We used paired student’s *t*-test to identify statistical differences in mean Intersection over Union (mIoU) for this dataset split.

Our attention module improved the original ResUNet++ by an average of 1% (*p* = 0.043), and the addition of transposed convolution further enhanced performance by approximately 1% (*p* = 0.031), as shown in Table 2. The AFNet model slightly increased network parameters but yielded a substantial performance improvement. All three networks’ dice loss scores remained low, with AFNet noT achieving the lowest loss score. However, our training results showed that many models with very low dice scores (<0.1) tended to overfit the test set, resulting in poor mIoU performance. Notably, our model achieved a significant increase in recall (5%), indicating reduced over-segmentation of background pixels at the expense of a slight decrease in precision (1%), leading to over-segmentation of amniotic fluid (AF). The improvement in recall was primarily due to the changes introduced by the attention module, allowing the residual block to focus on the background class of non-AF regions.

We compared the performance of our network to other state-of-the-art models, including U-Net [27], Double U-Net [30], DeepLabV3+ [29], ResUNet++ [24], and U-Net++ [28]. These networks were selected from top-ranking competitive medical segmentation challenges. The main metric for segmentation accuracy was mean Intersection over Union (mIoU). Our model demonstrated superior performance, significantly outperforming U-Net, DeepLabV3+, and Double U-Net regarding mIoU (*p* < 0.05; Table 3). Although the highest mIoU was achieved by U-Net++, our model showed no significant difference compared to it (*p* = 0.26). Regarding dice loss, AFNet scored lowest compared to U-Net, U-Net++, DeepLabV3+, and Double U-Net. Among all the models, Double U-Net achieved the highest recall and precision scores. However, when assessing these models, mIoU remained the most important metric for performance evaluation.

The results from Table 3 highlight the significance of attention blocks, ASPP, atrous and transpose convolutions in the segmentation of amniotic fluid (AF). Models that lacked these advanced modules underperformed and had more parameters, which hindered their ability to generalize well on the test set. The UNet++ model, with its increased number of skip connections, effectively reduced its capacity and mitigated the risk of overfitting. Overfitting is a common challenge in deep learning models, particularly when working with small datasets, as is often the case in fetal MR imaging applications. This highlights the importance of developing efficient models with fewer parameters and implementing mechanisms to enhance generalizability. Previous research has demonstrated that compact skip connections can improve the generalization of deep models without adding extra parameters [31].

Training deep learning models requires time for optimization, but from our analyses in Table 4, the number of parameters did not correlate with the training time due to early stopping. The larger models, such as UNet and Double UNet, converged around the same time as AFNet and UNet++, while DeepLabV3+ converged in a few minutes. These findings indicate that the model size does not solely determine the training time; other factors, such as optimization strategy and convergence behaviour, also play a role.

**Figure 4:**
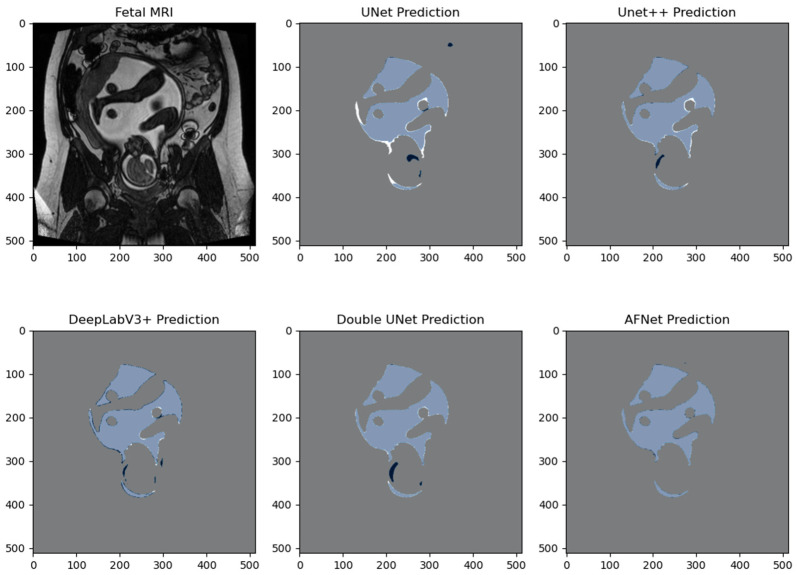
Pixel-wise comparison of segmentation masks. Coronal T2-weighted image of the gravid uterus. Prediction masks (dark blue) are overlaid with the ground truth mask (white). Light blue pixels signify a true positive overlap segmentation, dark blue pixels demonstrate a false positive segmentation, white pixels demonstrate a false negative segmentation, and grey pixels demonstrate a true negative segmentation.

**Figure 5:**
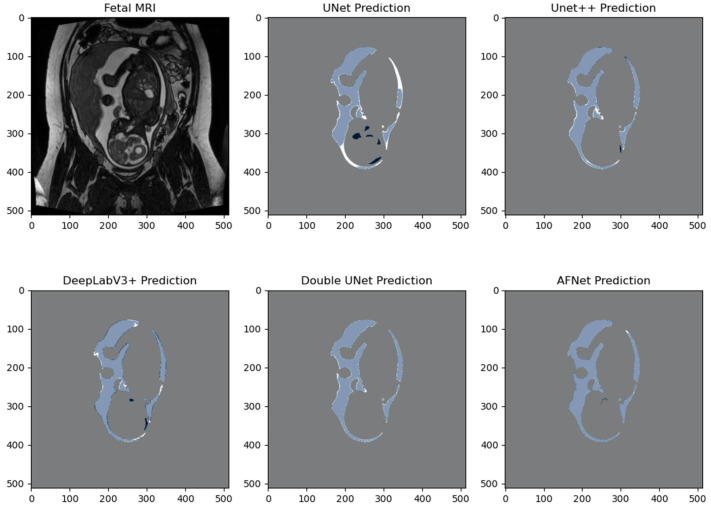
Pixel-wise comparison of segmentation masks. Coronal T2-weighted image of the gravid uterus. Prediction masks (dark blue) are overlaid with the ground truth mask (white). Light blue pixels signify a true positive overlap segmentation, dark blue pixels demonstrate a false positive segmentation, white pixels demonstrate a false negative segmentation, and grey pixels demonstrate a true negative segmentation.

We created pixel-wise visual comparisons to compare the performance of the various models from Table 3. The U-Net and Double UNet predictions exhibited over-segmentation of the AF, as indicated by the dark blue segmentation mask. In contrast, DeepLabV3+, UNet++, and AFNet produced segmentations of the amniotic fluid with high overlapping similarity. Figure 4 and Figure 5 illustrate these comparisons. It is important to note that these figures represent only a single slice and cannot fully capture the variability of 3D fetal MRI. Therefore, they offer qualitative insights into the areas where the models struggled. Most networks encountered challenges distinguishing AF from cerebral spinal fluid, eyes, oesophagus, bladder, and surrounding fat tissue. Moreover, they faced difficulties segmenting small crevices of AF and regions with intensity gradient boundaries. Introducing contrast augmentation in the training data proved beneficial for all networks, likely due to their reliance on small-intensity changes for AF delineation.

## 4. Conclusions

Our study presents a novel and unique approach to automated segmentation of amniotic fluid based on fetal MRI using the AFNet model, an improved version of the state-of-the-art ResUNet++ architecture. By replacing upsampling blocks with transpose convolutional blocks and average pooling layers with atrous convolutional blocks, AFNet demonstrates enhanced performance in AF segmentation. Our experiments revealed that utilizing Adagrad with a high learning rate was a more effective optimization strategy for this network. Furthermore, we observed improved performance by training usingwhole images with a smaller batch size instead of cropping.

The AFNet model and the corresponding dataset offer a solid foundation for further refinement and development to enhance its performance and expand its generalizability. While the performance improvements demonstrated in this study are based on our specific dataset, we believe the model exhibits robustness and reliability. However, it is important to acknowledge the limitations of this algorithm, such as the dataset size, inclusion criteria, demographic, unseen MR artefacts, MR acquisition slice, and the use of only T2-weighted MRIs. To establish its effectiveness across different datasets, future work should investigate its performance in other segmentation tasks, such as placental segmentation. Exploring more image post-processing and preprocessing techniques may further improve the segmentation results.

There are several avenues for future research to enhance the AFNet model. For instance, investigating the utilization of ASPP in earlier encoder layers could lead to performance improvements. Furthermore, incorporating transformer attention modules into the model may optimize attention blocks and enhance the model’s overall performance. Clinical evaluations of AF disorders could be conducted to establish correlations with neonatal outcomes, similar to the assessment of amniotic fluid index (AFI) and single deepest pocket (SDP).

Throughout our evaluation, AFNet demonstrated strong performance across various metrics and evaluation steps. It can perform 2D and 3D segmentations, making it suitable for analyzing 2D and 3D MR sequences. The ablation results highlighted the significantly improved recall of our proposed AFNet compared to the original architecture, indicating its enhanced utility as a clinical diagnostics tool.

In summary, our AFNet model significantly advances automated amniotic fluid segmentation based on fetal MRI. Its novel architectural modifications, competitive performance, and consistent results position AFNet as a valuable tool for accurate and efficient AF segmentation.

## Figures and Tables

**Table 1:** Confusion matrix, demonstrating subsets *S_o_*^1^ and *S_o_*^2^ where, *S_o_*^1^ + *S_o_*^2^ = *S_o_*_,_ which is the output segmentation set. Similarly, the subsets *S_g_*^1^ and *S_g_*^2^ represent the ground truth sets containing amniotic fluid and not having amniotic fluid, respectively.

Dataset	*S_o_*^1^ (Amniotic Fluid)	*S_o_*^2^ (Not AF)
*S_g_*^1^ (Amniotic Fluid)	TP	FN
*S_g_*^2^ (Not AF)	FP	TN

**Table 2:** Test results for baseline ResUNet++, AFNet noT, AFNet. The best results are shown in bold.

Model	Loss	mIoU	Recall	Precision	# of Parameters
ResUNet++	0.1305 ± 0.12	91.36% ± 2.7	90.56% ± 5.3	**93.66%** ± 1.4	**4.07 M**
AFNet noT	**0.1228** ± 0.042	92.46% ± 1.6	94.28% ± 1.2	92.46% ± 1.6	4.85 M
AFNet	0.1295 ± 0.078	**93.38%** ± 1.3	**95.06%** ± 1.2	92.01% ± 2.0	4.80 M

**Table 3:** Averaged test results of our AFNet with comparable state-of-the-art models. The best results are shown in bold. (* statistically significant, *p* < 0.05).

Model	Loss	mIoU	Recall	Precision
U-Net	0.5697 ± 0.14	80.04% * ± 3.4	93.65% ± 4.1	89.67% ± 2.3
UNet++	0.1668 ± 0.066	**93.65%** ± 0.70	96.00% ± 0.90	90.34% ± 0.80
DeepLabV3+	0.3939 ± 0.043	75.92% * ± 1.7	91.21% ± 1.7	90.26% ± 0.90
Double UNet	0.3288 ± 0.073	78.80% * ± 4.0	**97.24%** ± 0.33	**92.59%** ± 0.58
AFNet	**0.1295** ± 0.078	93.38% ± 1.3	95.06% ± 1.2	92.01% ± 2.0

**Table 4:** The number of parameters and average training time of models. The best metric is shown in bold.

Model	Training Time * (min)	# of Parameters
U-Net	48.0 ± 14.0	31.0 M
UNet++	50.0 ± 26.0	9.17 M
DeepLabV3+	**6.0** ± **3.0**	11.8 M
Double UNet	34.0 ± 33.0	29.2 M
AFNet	47.0 ± 18.0	**4.80 M**

* Training time signifies the time in which the model saved the best epoch, a model may have trained for longer but showed no improvement.

## Data Availability

The datasets presented in this article are not readily available because the hospital’s research ethics board does not permit sharing of clinical images. Requests to access the datasets should be directed to D.S., dafna.sussman@torontomu.ca.
